# The emergence of imaging mass spectrometry in drug discovery and development: Making a difference by driving decision making

**DOI:** 10.1002/jms.4717

**Published:** 2021-03-16

**Authors:** Stephen Castellino, Nichole M. Lareau, Mark Reid Groseclose

**Affiliations:** ^1^ GlaxoSmithKline Bioimaging Collegeville Pennsylvania 19426 USA; ^2^ Xenovista LLC Chapel Hill North Carolina 27516 USA

**Keywords:** drug development, drug discovery, drug efficacy, drug safety, MALDI imaging MS, medicine pipeline

## Abstract

The pharmaceutical industry is a dynamic, science‐driven business constantly under pressure to innovate and morph into a higher performing organization. Innovations can include the implementation of new technologies, adopting new scientific methods, changing the decision‐making process, compressing timelines, or making changes to the organizational structure. The drivers for the constant focus on performance improvement are the high cost of R&D as well as the lengthy timelines required to deliver new medicines for unmet needs. Successful innovations are measured against both the quality and quantity of potential new medicines in the pipeline and the delivery to patients. In this special feature article, we share our collective experience implementing matrix‐assisted laser desorption/ionization imaging mass spectrometry (MALDI IMS) technology as an innovative approach to better understand the tissue biodistribution of drugs in the early phases of drug discovery to establish pharmacokinetic‐pharmacodynamic (PK‐PD) relationships, as well as in the development phase to understand pharmacology, toxicology, and disease pathogenesis. In our experience, successful implementation of MALDI IMS in support of therapeutic programs can be measured by the impact IMS studies have on driving decision making in pipeline progression. This provides a direct quantifiable measurement of the return to the organization for the investment in IMS. We have included discussion not only on the technical merits of IMS study conduct but also the key elements of setting study objectives, building collaborations, data integration into the medicine progression milestones, and potential pitfalls when trying to establish IMS in the pharmaceutical arena. We categorized IMS study types into five groups that parallel pipeline progression from the earliest phases of discovery to late stages of preclinical development. We conclude the article with some perspectives on how we see MALDI IMS maintaining relevance and becoming further embedded as an essential tool in the constantly changing environment of the pharmaceutical industry.

## 1 INTRODUCTION

The successful implementation of a new technology in pharma or a biotech can be challenging even with a very well‐developed value proposition. With the cost of R&D in the billions of dollars and the timelines typically in excess of 10 years, pharmaceutical discovery and development strategies continue to be closely scrutinized for opportunities to improve efficiency and effectiveness.^1^ Close attention is paid to understanding the decision‐making processes of successful medicines with a specific focus on how the timelines could have been reduced. Furthermore, attrition metrics are analyzed in an effort to improve the drug discovery process with the ultimate goal of increasing the probability that drug candidates selected for development will become medicines.^2^, ^3^, ^4^ Although innovation and decision making are critical to the success and survival of pharma and biotech firms, the choices of which paradigm changes to pursue or technology investments to make are not a simple task. The individuals making these decisions at various levels of the organization often have a bias based on their backgrounds and can be skeptical of a paradigm shift based solely on a new analytical technology. Considerations are not only for the cost of instrumentation and related facility upgrades but also for the internal expertise to successfully deliver expectations and positively impact pipeline progression. When investigators do achieve funding, the approval process can sometimes create inflated expectations or unrealistic timelines making successful implementation difficult. It is important for the scientific investigator initiating a new analytical technology like imaging MS to understand that success is directly tied to impacting the discovery and launch of new medicines. This can be achieved by generating data and information that confidently drives key decision making and milestones for drug candidate advancement. Analytical chemists must be willing to expand their scientific knowledge of the requirements and milestones for drug candidate progression from discovery to the clinic. Efficiently adapting best strategies, practices, and methods associated with the new technology can avoid some of the pitfalls and lengthy delays common to a trial and error approach. Thus, the aim of this special feature article is to share our collective experience in designing, conducting, and delivering impactful results using matrix‐assisted laser desorption/ionization imaging mass spectrometry (MALDI IMS) in the preclinical space of pharmaceutical discovery and development.

**FIGURE 1:**
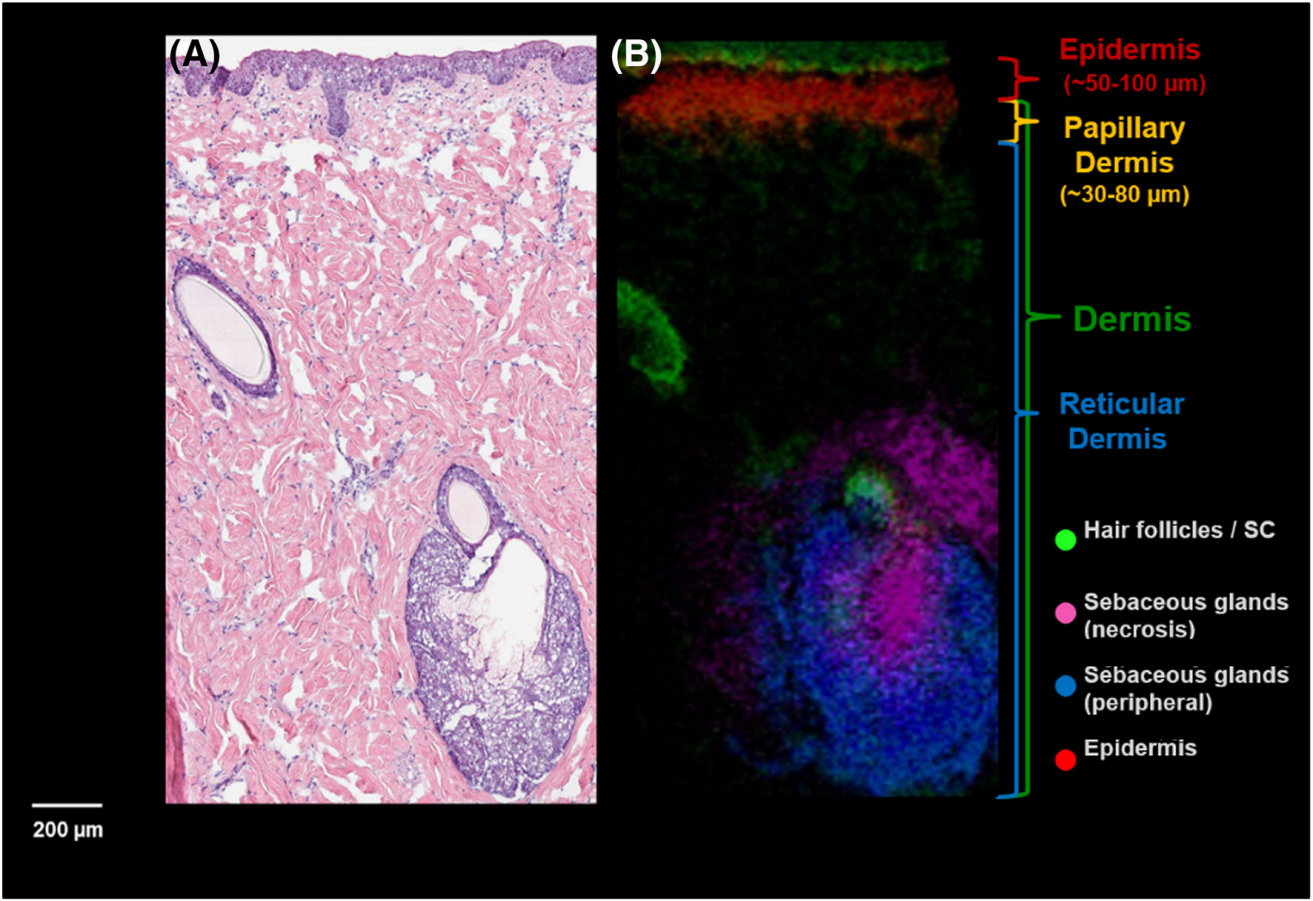
An example: co‐registration of endogenous markers from MALDI IMS and the corresponding H&E image. (A) The H&E stained skin tissue section showing the various regions from the epidermis to the dermis. (B) The MALDI IMS image depicting multiple endogenous ions that highlight specific histological features of the skin such as hair follicles and sebaceous glands. The combination of the histological detail from the stained image and the molecular information from the MALDI IMS assists in co‐registration and anatomical assignment (Figure courtesy of Jeremy Barry and Fang Xie, GSK Bioimaging, USA)

At its core, IMS is an ideal tool to determine the in situ quantitative biodistribution of analytes in tissues or cellular matrices. Currently, most IMS studies conducted in drug discovery and development focus on the quantitative distribution of drug candidates and their metabolites in animal model tissues.^5^, ^6^ These studies may also include known endogenous molecules that can serve as “molecular” histology markers to contextualize drug localization and facilitate co‐registration of ion images with serial histology tissue sections (Figure 1). In addition to studies that are solely focused on the analysis of drug‐related material are study designs that are non‐targeted such that there is no a priori list of molecular species for analysis. These study designs are emerging and have not been regularly incorporated in drug discovery or development. The goal of a non‐targeted approach is focused on identifying changes in endogenous molecular distributions due to pharmacology, toxicity, or disease pathogenesis relative to a control. The success of these studies could lead to characterization and/or validation of molecular pathways, mechanisms, and biomarkers of both pharmacology and toxicology. Furthermore, these studies can be used to identify pathways associated with the disease state and ultimately create molecular images of tissues where histological features are represented by molecular species.

The need to understand the biodistribution of drugs in tissues is not a new concept, but historically, the analytical tools available to achieve this were limited. The introduction of IMS has addressed this gap by providing the ability to quantitatively characterize the distribution of drugs within tissues providing direct evidence of whether the drug is reaching the intended target. Routinely in drug discovery and development, this critical element of establishing efficacy of a pharmacologically active compound is based on indirect measurements. These include the use of plasma centric approaches where it is assumed that the plasma pharmacokinetics (PK) is predictive of the targeted tissues containing the intended active site. Alternatively, homogenates are used to assess tissue concentration levels. This method assumes that organs could be treated as single compartment boxes where a drug is homogeneously distributed in the tissue. IMS provides a means to go beyond these approximations and directly assess the distributions within tissue compartments and collect PK data based on targets within the tissues and not plasma alone. This is not to say that plasma PK measurements, estimating “unbound” drug levels, or even evaluating tissue homogenates concentrations have no place in the fast‐paced drug discovery and development arena. Rather, we have determined that IMS is the best quantitative technology for determining the drug distribution in tissue sub‐compartments. IMS tissue distribution data can also be used to calibrate and contextualize, often quicker, plasma centric approximations as well as enhance modelling and simulations. Furthermore, as we are compelled to understand and investigate smaller discrete functional regions of organs including cell clusters, the need to understand pharmacology, disease pathogenesis, and adaptive responses in these foci will be necessary. Thus, the “omics” tools, now broadly applied across whole organs, will become tailored to “spatial omics” and be integrated further into the spatial drug or biomarker distributions established by IMS.

Although spatial omics is an exciting new frontier, we want to stress the importance of quantitative IMS in drug discovery and development. Early in our experience while employing qualitative IMS studies, the feedback was that the impact in driving project decision making would be limited without quantitative data. Qualitative IMS experiments can provide value when the project decision is solely based on the observation of the drug candidate being present or absent at the target site; however, a cornerstone of drug discovery is understanding the quantitative relationship between drug dose and the desired therapeutic effect. This includes a quantitative understanding of drug disposition, which entails absorption, distribution, metabolism, and excretion (ADME). At the target tissue level, we need to understand the relationship between the amount of dosed drug that reaches the target and the desired therapeutic response that results in reestablishing homeostasis. It is also necessary to understand the quantitative relationship between the amount of dosed drug that can initiate toxicity at the intended or unintended target tissues. Therefore, if you want to conduct biodistribution experiments that will drive program decision‐making, quantification must be a part of the deliverable.

Fortunately, quantitative MALDI IMS methods have evolved and are well established in the literature with numerous examples of the impact ranging from discovery to the clinic.^7^, ^8^, ^9^, ^10^ Furthermore, validation of IMS quantification can also be supported by traditional homogenization LC–MS, laser capture microscopy and subsequent MS analysis, or in some instances surface liquid sampling—followed by ESI measurements for the most critical decision‐making studies.

## 2 PLANNING FOR SUCCESS!

The organizational structure within pharma can be complex. Typically, unmet medical needs are identified, and a project team of scientists and managers are assigned to serve as the stewards of discovering, developing, and delivering a safe and efficacious medicine. The autonomy of these project teams within different pharma organizations will vary, and the size and membership may also change depending on the stage within the pipeline, but a core group of scientific leaders will remain on the team from inception through delivery to patients. Although the project team is responsible for strategies, planning, and decision making, study execution is conducted by internal scientific departments or external contract organizations. There is an extensive “matrix” organization that exists between project teams and the internal scientific “support” teams that have different line managements. Thus, managing resources, expectations, and funding for IMS groups can be challenging. Based on our experience, the delivery of impactful IMS study outcomes that are recognized as essential by project teams is necessary for long‐term viability and stability of the IMS group.

The measure of success for any IMS study in pharmaceutical discovery and development is based on study outcomes playing an integral role in the project team's decision to progress the program to the next milestone or follow remediation which could include termination. Well‐defined objectives are essential in driving decision‐making. This is where communication between the IMS leads and the project team cannot be taken for granted. It is key to clearly explain all possible IMS study outcomes to a project team and set well‐defined objectives. It is also extremely helpful if you understand the program background, stage in the pipeline, and current milestones. As project team members are immersed in all aspects of the program with dialog occurring primarily between themselves, communication of all the details necessary for an optimum IMS study to scientists outside of the project team may be overlooked. In these instances, IMS team members must be able to address the knowledge gaps and even raise important points to consider in the study design for the collaboration to be successful. Furthermore, the study outcomes should integrate previously collected data (in vitro pharmacokinetics [PK] and pharmacodynamics [PD]) to forecast strategies for future studies (development; the clinic).

**FIGURE 2:**
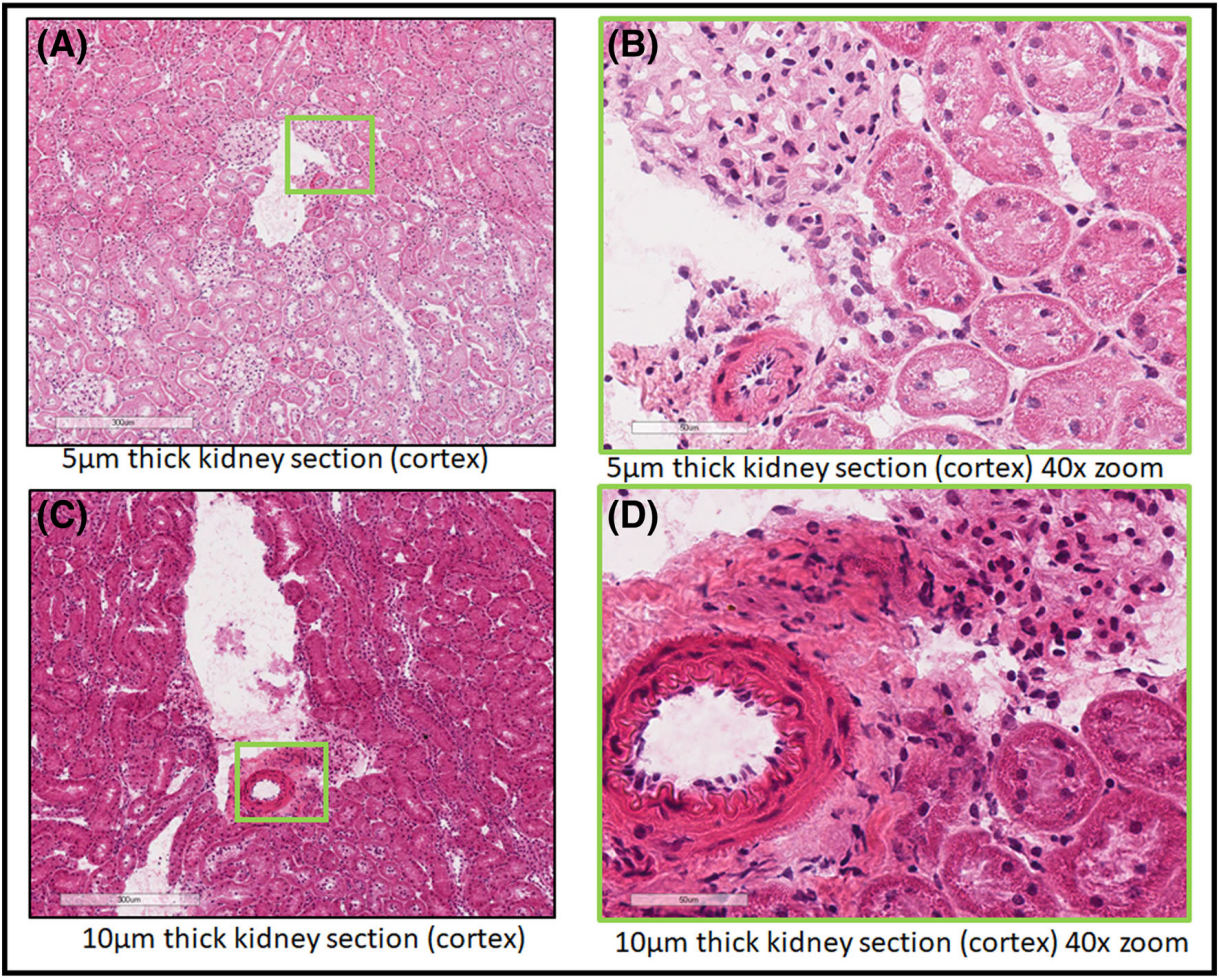
A comparison between two frozen tissue sections of rat kidney tissue cut at different thicknesses. (A) Rat kidney tissue sectioned with a 5 μm thickness followed by H&E staining. A zoomed in region of the cortex is annotated with a green box indicating the region displayed in Panel (B). (B) A region of the cortex of the rat kidney at 40× magnification showing in focus nuclei and tubule linings. (C) A section from the same rat kidney as above. This section was collected with a 10 μm thickness and H&E stained. A region of the cortex is annotated by a green box as the region shown in Panel (D). (D) A region at 40× magnification that shows the artifacts due to thicker tissues sections

MALDI IMS is an analytical platform that engages all disciplines in the pharmaceutical discovery and development pipeline, from chemists and biologists to pathologists, clinicians, and modelers. Dialog with the various project scientists early on can be critical in achieving IMS study success. We established a close working relationship with our pathology department. This relationship has been essential in assessing and annotating tissue histology to integrate and interpret this information within the context of the IMS data. However, we learned that obtaining good H&E serial sections was necessary to receive critical insight into the histology, as pathologists retain a high acceptance criterion for section quality. In this regard, it is important to think not only about the IMS spatial resolution but also about the “clarity” of serial H&E images that will be co‐registered with molecular ion images.^11^ For instance, sectioning frozen tissue blocks at 10 μm versus 5 μm is much easier as tissues are typically not embedded. However, for most tissues, the H&E histology analysis at 5–6 μm is not as confounded for interpretation and analysis compared with 10 μm. Figure 2A,B shows a rat kidney sectioned at 5 μm thickness. Figures 2C,D shows the same rat kidney sectioned at 10 μm thickness. The left‐hand panels show the cortex of the kidney where the tubules and other substructures are more defined in the thinner section (Figure 2A at 5 μm) versus the thicker section (Figure 2C at 10 μm). The right‐hand panels show zoomed regions at 40× magnification to better visualize the clarity differences in relation to tissue section thickness. Because multiple cellular layers compose a tissue section at 10 μm thickness, the borders of the substructures appear to have a blurred effect (Figure 2D) compared with the thinner section at 5 μm (Figure 2B). These effects of the multicellular layer distortion cause a similar blurring in higher spatial resolution IMS images (~10 μm spatial resolution) as well. Therefore, the histological detail and morphological specificity necessary to contextualize the IMS images, especially for high spatial resolution, must be taken into consideration even though this may mean spending more time hunched over the cryostat.

We have found that having limit of detection (LOD) estimates for the drug candidate by MALDI IMS is a critical starting point for any discussion with a project team. It is important to know if the planned preclinical study will have tissue concentrations at or above the IMS LOD to enable the study. Exposure levels achieved in the preclinical IMS study must be relevant to the clinical therapeutic dose range to generate meaningful results for project decision making. Dosing to exposure levels in tissue higher than the therapeutic range to accommodate IMS detection can result in changes in drug disposition due to potential saturation of some pathways and in a worst‐case scenario trigger toxicity. There are instances where the tissue concentrations of therapeutically relevant exposures will not be amenable to a meaningful MALDI IMS analysis despite optimization; however, it has been our experience that with judicious optimization and study parameters, the biodistribution of most small molecule drug candidates can be assessed. We have found that for purposes of rapidly estimating the LOD of a compound, the tissue serial dilution method works well.^8^ Therefore, once you have an estimated LOD, you can assist the project team in selecting a dose level that is on the high end of the therapeutic dose range to ensure you have enough sensitivity to observe a reasonable dynamic range of distribution within the tissue. This will also provide some flexibility with selecting the spatial resolution.

The next detail to consider is tissue collection time points. If plasma PK data exist, these can be used as initial approximations for selecting which dose time points are within the IMS LOD. It is important to note that because an IMS study requires tissue collection, teams will often by default select the terminal PK time point for tissue harvesting, which is typically 24 h after the last dose. Because most drugs have half‐lives well below 24 h, there could be little drug present in the tissue 24 h after the last dose despite a large initial dose. The exception being is if there is accumulation of the drug or metabolites after repeat dosing. There are several strategies that can be used to deliver both PK and biodistribution by IMS objectives within the same study. One approach in the discovery phase is to complete the dosing (single or repeat) and plasma sampling requirements for the PK portion of the study through the 24 h time point and then administer one additional dose prior to harvesting the tissue at an optimal time point for IMS. This strategy has the advantage of reducing animal use but will require additional amounts of the drug candidate. Alternatively, in addition to the PK group, a separate group of animals can be used for the MALDI IMS objectives, but it is important that adequate PK sampling from both groups is completed to provide a bridging between the IMS and PK groups.

Typically, sample collection at the maximum drug concentration (C_max_) will be optimum for IMS analysis, which can be estimated by plasma PK data. However, there are several circumstances where a much later or earlier time point might be considered based on the specific IMS study objectives. Whenever possible, control animal tissues should be included in the IMS study to determine if interfering endogenous peaks could confound drug detection and quantification. Additionally, a control tissue comparison is critical when evaluating endogenous molecular changes occurring because of treatment or disease. The IMS analysis of control tissues can also serve as a quality check by demonstrating that misdosing did not occur during the study. Although time course study designs can be the most informative and may prove necessary in certain instances, such as demonstrating causality in toxicology studies, these studies tend to be limited to more critical endpoints in later stage development because of the higher cost due to an increased number of animals and resources.

One of the biggest challenges in early drug discovery is delivering information on a time scale relevant to the team. One way to think of this discovery timescale is to use the rate of synthesizing a new candidate molecule, which can be on the order of days with modern synthetic chemistry methods. Therefore, IMS studies at this stage must have well‐defined objectives with relatively short deliverable timelines. If studies become protracted due to the design or meandering objectives, the IMS data may have little impact as the team may have already moved on to other molecular scaffolds or progressed to the next milestone by the time the IMS data are available. The size of the study design must also be carefully managed with a focus on the primary objectives needed to progress the program versus exploratory endpoints, which might provide additional supportive information. For instance, if considering three dose groups plus a control group with a minimum of three animals per dose group and a single target tissue, the IMS portion entails 12 tissues to sample without taking into consideration the tissue section replicates from each block. An alternative strategy that can be effective is a study design that utilizes several compounds. Although these studies may take longer to complete, the data can still be relevant regardless of shifting drug leads because they define the relationship with molecular physicochemical properties with biological biodistribution and form a framework for the program.

Another consideration is the spatial resolution necessary to achieve the study objectives. As this parameter can affect the sensitivity (LOD), some forethought as to the species, tissue heterogeneity, and the histological subcompartment resolution required is needed. For instance, brain tissue is relatively heterogeneous, and subcompartments such as the cerebellum, pons, and ventricles are readily distinguished with spatial resolutions ≥150 μm. On the other hand, distinguishing the zonal distribution in liver tissue may require pixel dimensions **≤**25 μm. A study strategy that we have found effective is first acquiring a “survey” image of the drug distribution within the tissue at a lower spatial resolution with optimal sensitivity for quantitation, followed by higher spatial resolution imaging on a serial section of subregions of high localization or specific relevance to the drug target.

**FIGURE 3:**
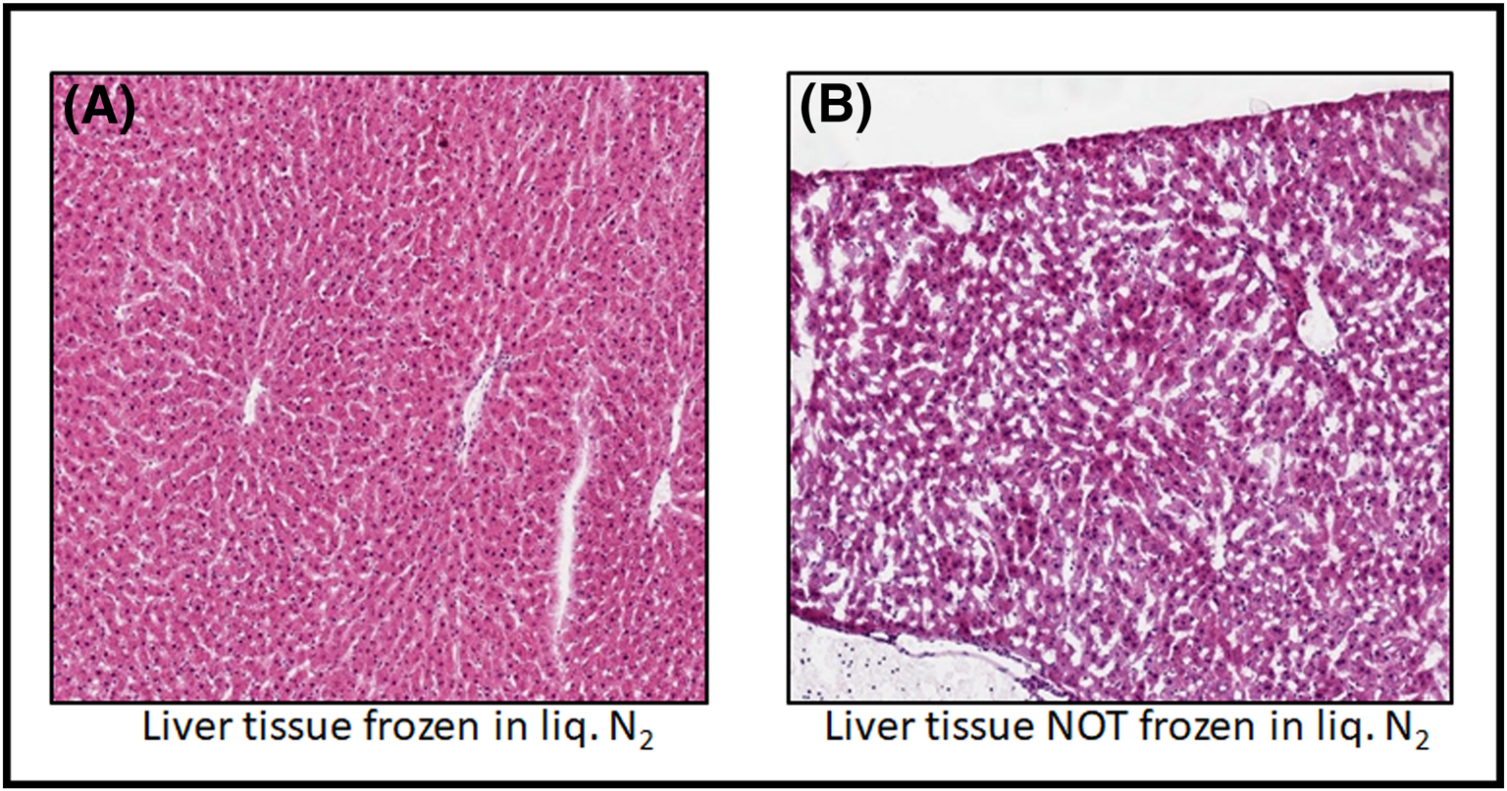
An example comparison between two sectioned frozen liver tissues after different freezing methods have been applied. (A) An H&E stained liver tissue section from a liver lobe that was frozen using liquid nitrogen. (B) An H&E stained tissue section from a liver low that was not frozen in liquid nitrogen (frozen on dry ice) highlighting freezing artifacts that arise from a slow freezing process

It has been our experience that one of the most critical elements of the study is tissue sample collection at necropsy. This is especially true if the tissues are being collected at a contract research organization (CRO) that has little experience in tissue collection for IMS analysis. The morphology and molecular integrity of excised tissues needs to be maintained prior to flash freezing. This allows the analysts to select the most meaningful sectioning planes for analysis, more accurately identify where the IMS planes were acquired from using a tissue atlas and provide meaningful histology images. Therefore, tissues should be properly laid out and not compressed or distorted by the packaging method. Tissues must be flash frozen for optimum histology. A slow freeze results in the formation of ice crystals in the tissue and will translate to “holes” or gaps in the corresponding histology images as shown in Figure 3. Liquid nitrogen cooled isopentane or liquid nitrogen are best for flash freezing tissue with minimum freeze artifacts. The larger the tissue block, the greater the chances of uneven freezing and fractures, therefore, consider reducing the size of the tissue block. Tissue block orientation with regard to the original organ should also be included for the purpose of mapping individual tissue sections back to the original organ. For brain tissue, taking advantage of the rodent brain atlas references as well as sectioning distance from the bregma in coronal sectioning are useful tools for locating imaging planes into the whole organ.^12^ One final note, if samples are to be shipped after storage at −80°C, make sure that the samples are packed appropriately to prevent them from being fractured due to handling and motion of shipment with dry ice. More than once, we received samples that need to be “assembled” after arrival. The best way to ensure proper tissue alignment and freezing is to collect and process tissues yourself when possible. Other general sample preparation considerations such as matrix application can be obtained from numerous literature reviews and articles.^5^, ^6^, ^13^

## 3 STUDY CONDUCT

### 3.1 Complex in vitro models

Cell‐based screening assays are a key component of the early stages of drug discovery and provide valuable information on potency, pharmacology, and cytotoxicity.^14^ Historically, 2D monolayer cultures have been the predominate cell‐based assay due to their simplicity and amenability to high‐throughput screening applications, where many different compounds and conditions are tested in parallel to identify potential lead compounds for investigation. However, the simplicity of these systems also underlies their major limitation: the inability to mimic in vivo cellular behavior. Although cells can survive in this rigid 2D environment, it has been shown that this unnatural environment affects signal transduction, gene expression and cellular behavior, and therefore, cells may lose their original phenotype or specific function.^15^ This recognition has led to the development of increasingly sophisticated 3D cell cultures to more accurately reproduce the complexity of an in vivo environment. Using scaffolds, hydrogels, and microfabricated devices, 3D cell culture technology has rapidly developed as a promising assay for testing of drug delivery, metabolism, pharmacology, and toxicity.^16^ MALDI IMS has the potential to be a key part of the analytical strategy for assessing 3D cell cultures by measuring drug penetration, distribution, and cellular response to the drug and other stimuli. The ability to assess multiple treatment conditions across several time points on a well‐controlled, yet biologically relevant, sample is highly attractive. This type of sample could provide the opportunity to elucidate subtle mechanistic changes that get overshadowed by the inherent variability of in vivo studies.

**FIGURE 4:**
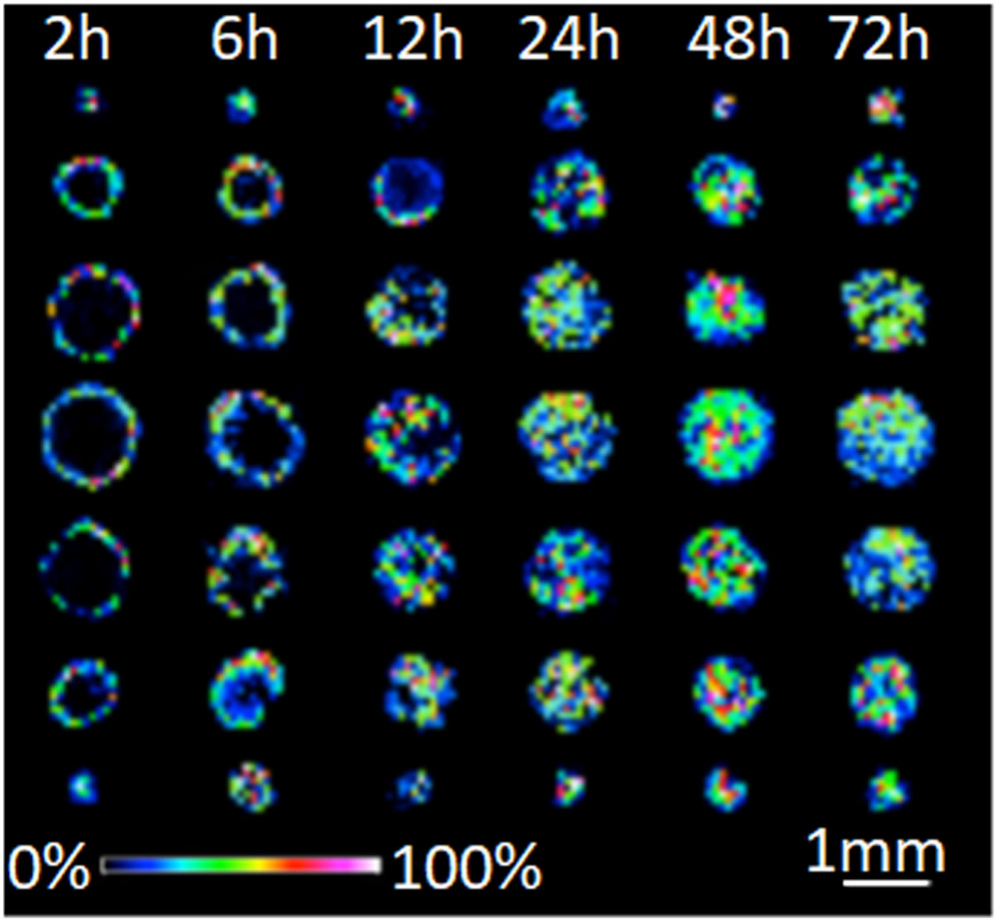
Time‐dependent penetration of irinotecan (m/z 587) in HCT 116 spheroids analyzed by MALDI‐IMS. Spheroids were treated with 20.6 μM irinotecan for 2, 6, 12, 24, 48, and 72 h (from left to right). For every treatment duration, color gradient intensity maps were generated from seven consecutive 12 μm slices from a single spheroid in 120 μm vertical intervals. Reprinted (adapted) with permission from Liu, X., Weaver, E. M., Hummon, A. B., *Anal. Chem*., 2013, 85, 6295–6302. Copyright 2013 American Chemical Society

Several methods for the application of IMS to 3D cell cultures have been reported including evaluation of drug penetration and cellular response in spheroids^17^, ^18^, ^19^, ^20^, ^21^ and organoids.^18^, ^22^, ^23^ For example, Liu et al. used MALDI IMS to investigate the time‐dependent penetration of irinotecan at several different concentrations in HCT 116 spheroids (Figure 4).^24^ Additionally, three metabolites of irinotecan were identified and shown to be localized to the outer rim of the spheroids, possibly indicating increased metabolism in the viable cells at proliferative and quiescent zones.

One major challenge in the analysis of 3D cell cultures by IMS is the sample preparation steps required to facilitate imaging analysis. For example, spheroids/organoids grown in 96‐ or 384‐well plates must be individually transferred to an embedding mold and embedded in a suitable matrix (e.g., gelatin) for freezing. This is a labor‐intensive, manual process, and the organoids/spheroids are not uniformly distributed across the *z*‐axis of the embedding mold. As a result, one must rely on visual inspection of the sample block containing the cell culture as it is sectioned to recognize when the appropriate *z*‐axis plane is reached. Johnson et al. recently described a sample preparation strategy using a gelatin microarray microwell mold to align multiple organoids in the same axis.^23^ This type of innovation will be critical to help remove the sample preparation bottleneck and leverage the high‐throughput capabilities of IMS for analysis of 3D cell cultures.

Although further refinement of complex in vitro models and other platform technologies will continue to draw attention and resources in the pharmaceutical industry, new analytical approaches such as IMS are needed to analyze the pharmacology and toxicology in these systems and may also serve as bridging data for preclinical in vivo studies. The potential to generate human disease‐relevant in vitro models and thus reduce animal usage and study timelines is highly attractive; however, detailed understanding of the biological mechanisms and adequate validation of the clinical translation will be essential. Although the 3D cell culture platform is still evolving, we are following the developments closely and working with the complex in vitro model team to provide input and test various strategies for compatibility with MALDI IMS. Once the sample preparation challenges are overcome, IMS should be well‐suited to efficiently screen these systems for both drug distribution and endogenous molecular changes associated with treatment or disease phenotype.

### 3.2 Discovery: Drug at target

Drug candidate failure due to lack of efficacy in clinical trials has created a mandate in the discovery phase to establish a clear PK‐PD dose relationship and mechanistic understanding for the mode of action for all drug candidates. This has brought attention back to the “three pillars” of drug discovery that requires that the drug candidate (i) is reaching the target, (ii) engages the target, and (iii) creates a measurable and mechanistically relevant PD effect.^25^ Furthermore, assumptions and approximations associated with relying solely on plasma PK data have been challenged, as has relevance of the selected animal models. IMS provides a direct quantitative measure of “pillars” (i) and (iii) in animal models. In the discovery phase of research, it also can provide differentiation between several drug candidates based on chemical class, physiochemical properties, assay potency, metabolic stability, and potential off‐target effects. Importantly, IMS data from an in vivo model can serve as a calibration and refinement for in vitro measurements and simulations such as physiologically based pharmacokinetic (PBPK) modeling.

For the purposes of illustration, we highlight an example of a typical IMS study conducted during the drug discovery phase targeting neurological injury. The project team was interested in determining if the potential drug candidate could reach the target in the brain. The presence of the blood–brain barrier (BBB) can limit brain parenchymal access of systemically circulating therapeutic agents due to the tight endothelial junctions of blood capillaries. Additionally, active efflux transporters dominated by P‐glycoprotein (Pgp and ABCB1) but with contributions from breast cancer resistance protein (BCRP) and the multidrug resistance‐associated proteins (MRP1, MRP2, MRP3, MRP4, and MRP6) can further limit CNS penetration.^26^, ^27^ And although in vitro measurements (permeability and efflux ratio) and modeling can provide some basis for predicting drug candidate penetration into the brain parenchyma, the translation to an in vivo system can often be inaccurate or confounded. The direct quantitative measurement of drug brain penetration and distribution as a function of dose, administration routes, and PK is necessary to demonstrate that the drug candidate is reaching the target and is providing a basis for building an accurate PK‐PD relationship.

Traditionally, brain penetration was experimentally determined by combining brain homogenate LC–MS measurements with plasma PK to determine a brain to plasma ratio. If the ratio is greater than approximately 0.03–0.05 in the rodent model, it is assumed that there is some brain parenchyma penetration, and if the ratio is less than that, it is attributed to residual blood volume in the brain.^28^, ^29^ In contrast to the brain, liver to plasma ratios can be as high as 10–20 for some drugs.

**FIGURE 5:**
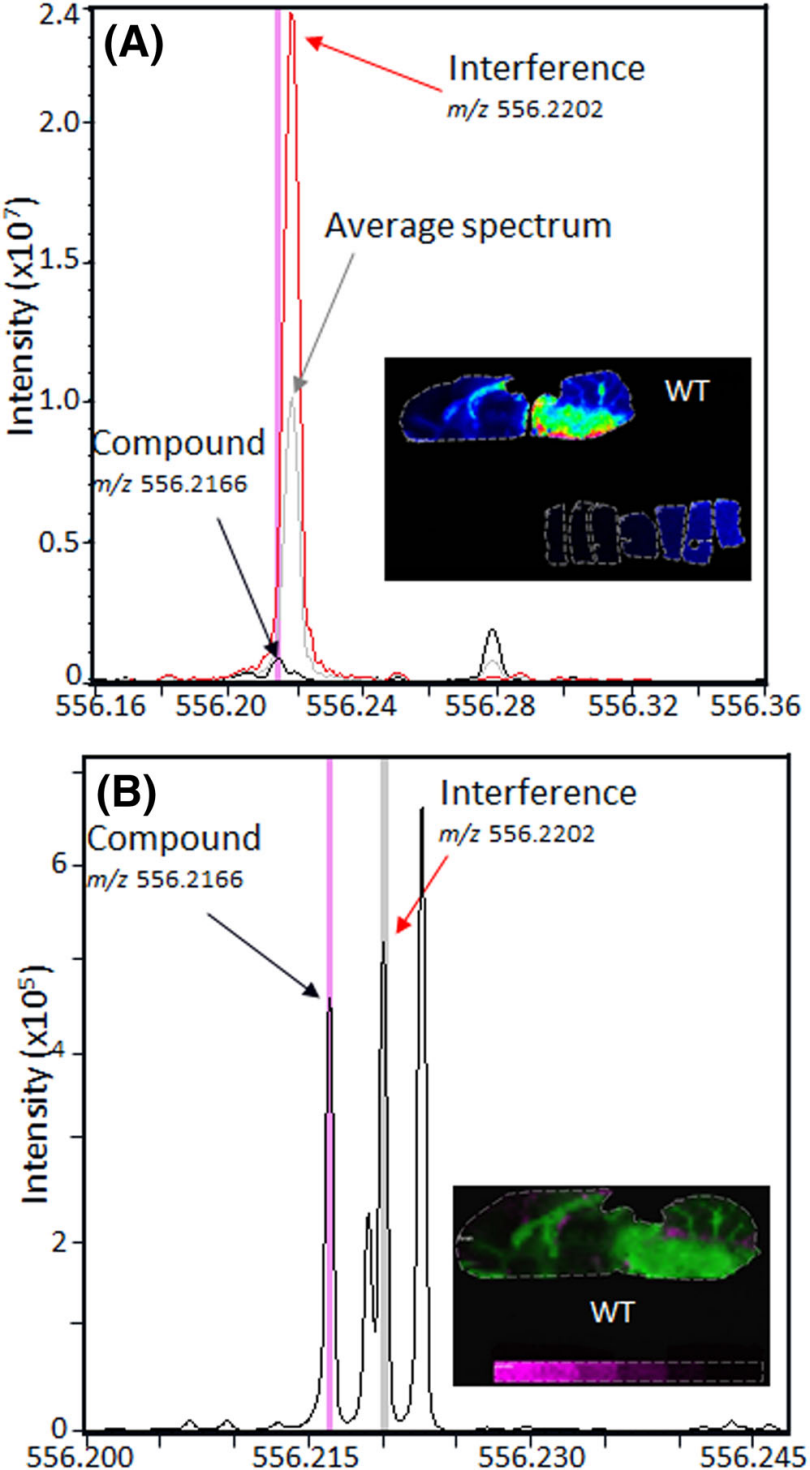
An example of the potential challenges that interfering endogenous ions can pose for quantitation and the ability to modify methods by increasing mass resolution to separate those interferences. (A) The spectral overlay from the ion image (inset) of the average spectrum (gray), mimetic model spectra (black), and spectra from the region in the brain of high interference (red). Due to the high abundance of the interfering ion, the compound of interest is not separated and cannot be accurately quantified. (B) The average spectrum from the ion image (inset) acquired with optimized acquisition methods to increase the mass resolution from 131 K resolving power (Spectrum A) to 1.4 M resolving power (Spectrum B) at m/z 556.2166. The modified method fully resolves the compound (pink) from the interference (gray) allowing for quantification of the compound in the image

In this example cited above, preliminary LC–MS brain to plasma ratios in the wild‐type (WT) rat model ranged from 0.2–0.35 depending on dosing delivery design, suggesting reasonable BBB penetration, consistent with the in vitro permeability and efflux measurements. The IMS study was designed to assess the distribution of the compound in the parenchymal brain tissue after intravenous (IV) infusion. The tissues were collected at the end of infusion without delay along with plasma PK data for each animal. Tissues were split along the midline of the brain such that one hemisphere was flash frozen for IMS and one for homogenization and LC–MS analysis. Initial IMS method development was conducted using a liver homogenate tissue mimetic model to assess the LOD for study design.^7^ As the first rat brain tissue was imaged along with the mimetic model, it became clear that there was an endogenous interference ion specific to the brain tissue. The resulting image with the interfering endogenous ion is displayed in Figure 5A,B. The comparison of the of the high‐resolution mass spectra from the mimetic model and the brain image highlights the interference. To separate these peaks, an extended acquisition time was required to increase the mass resolving power from 131 K to approximately 1.4 M at the peak of interest (556.216 Da). Although this required longer acquisition times, the resulting data clearly separate the interference and lower both the limit of blank (LOB) and the limit of detection (LOD) (LOB changed from 1.85 to 0.10 μg/g). With increased sensitivity and confidence in the quantitation measurement, three rat brains were analyzed, and the concentration of the compound was determined for regions of parenchyma, as well as blood vessels and ventricles.

**FIGURE 6:**
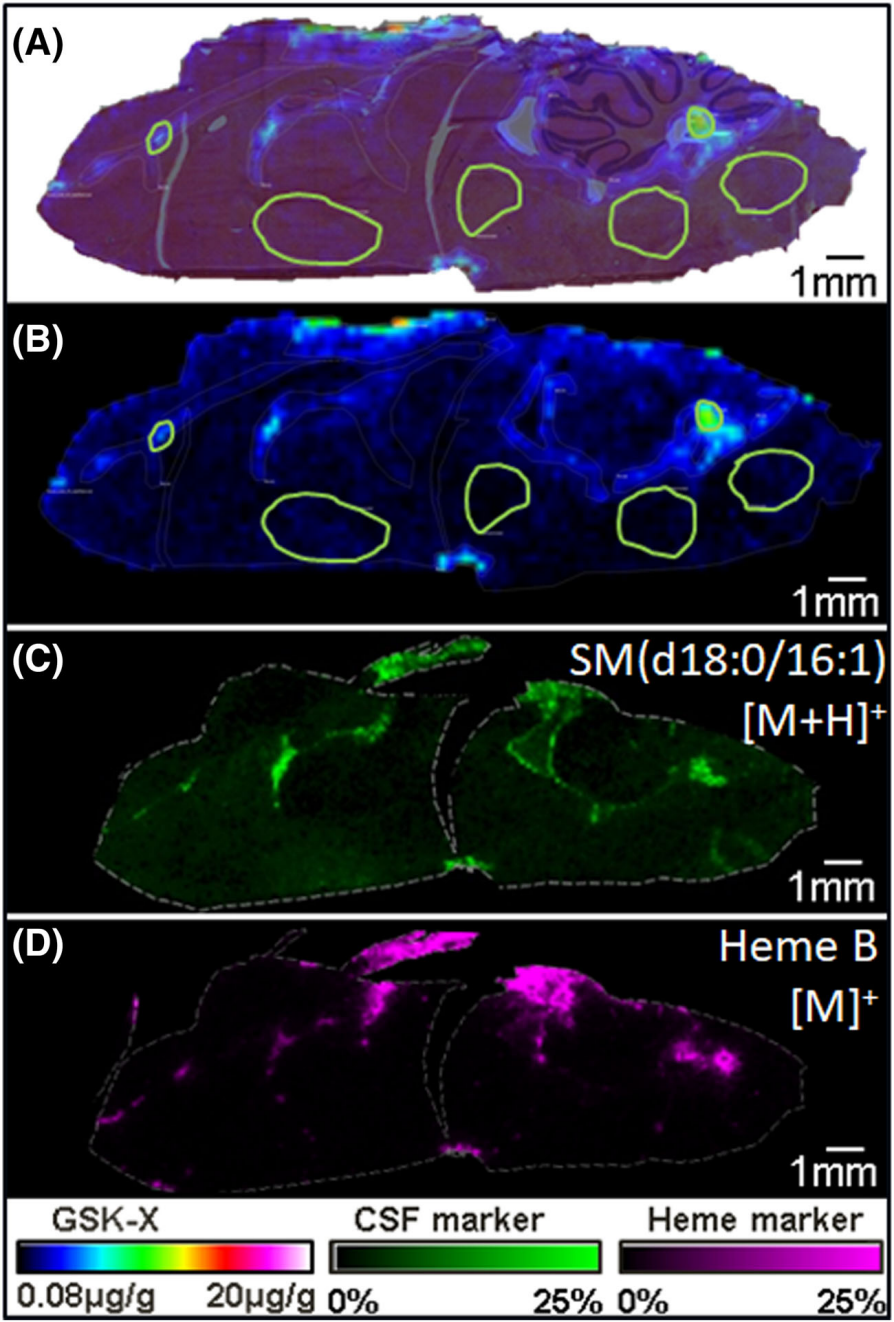
A multiplane image showing the ability to use endogenous markers to annotate anatomical regions in the brain to select ROIs for quantitation. (A) An ion image of the compound GSK‐X overlaid on the H&E stained rat brain with regions of interest marked in green. (B) The ion image of GSK‐X scaled from the LOB to 20 μg/g in rainbow color scale. (C) The ion image of an endogenous marker of the CSF in green from 0% to 25% absolute intensity. (D) The ion image of Heme B in pink as scaled from 0% to 25% and used to mark regions of vasculature. Using (C) and (D) to mark the CSF and vasculature, regions of blood vessels, ventricles, and parenchyma were annotated in Panels (A) and (B)

Endogenous ions were used as molecular markers for major blood vessels and for the cerebral spinal fluid (CSF) to denote ventricles. Mapping these regions of the brain allows for the selection of regions of interest (ROIs) specific to the parenchymal tissues. These ROIs allow for quantitation of the dosed compound while minimizing the contribution from the vasculature system. The mapping of endogenous markers and the selected ROIs of parenchymal tissue are displayed in Figure 6A–D. The ion signal of the compound appeared in multifocal areas in high levels throughout the brain tissues but was not localized to specific regions. Using the average concentration from the ROIs in each brain, a quantitative value was determined to describe the amount of compound that penetrated the brain. This study showed that the concentration in the parenchyma was not as high as predicted based on previous homogenate brain to plasma ratios. Additionally, the brain to plasma ratio for each rat was used to calculate an average brain to plasma ratio (average the whole tissue section for homogenate comparisons) as well as brain parenchyma to plasma ratio based on the selected ROIs. The high drug candidate concentration in the ventricles (~7 μg/g) suggested that efflux transport could be a major limiting factor for brain penetration and biasing homogenate measurements.

**FIGURE 7:**
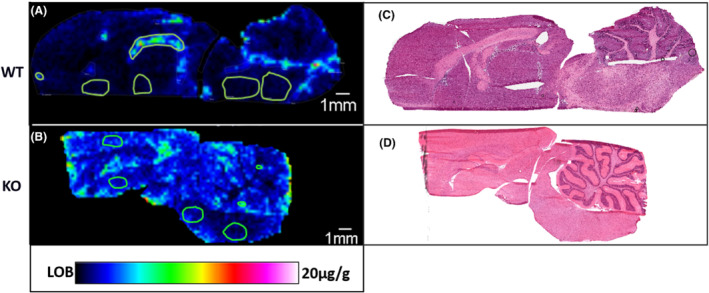
A comparison between the wild type and Pgp knockout rat brained after dosing with GSK‐X under the same conditions. (A) The ion image of GSK‐X in the WT rat brain scaled from the LOB to 20 μg/g in rainbow color scale with regions of blood vessels, ventricles, and parenchyma annotated in green for quantification. (B) The ion image of GSK‐X in the Pgp‐KO rat brain with regions annotated and scaled from the LOB to 20 μg/g in rainbow color scale. (C) The post‐MALDI acquisition stained H&E stained image of the tissue in Panel (A) to the left. (D) The H&E image of the tissue in Panel (B) stained after MALDI acquisition

The project team requested a follow‐up study using Pgp knockout (KO) rats to better understand the extent to which efflux transport and Pgp specifically was responsible for limiting brain penetration for this compound. A figure comparing the distribution of the dosed compound in the WT and KO brain is displayed below in Figure 7A,B. A 3.5× increase in the compound concentration in the parenchymal tissue was also observed in the Pgp KO dosed brain compared with the parenchyma of the WT dosed brain. The corresponding H&E images of the brain tissue sections are shown in Figures 7C,D. MALDI IMS data were further validated by using the non‐imaged hemisphere of the brain for homogenization and LC–MS. The homogenate LC–MS quantitation values were in good agreement with the MALDI IMS data.

The quantitative tissue distribution by IMS gave the team a true assessment of the amount of the drug candidate reaching the targeted brain parenchyma as a function of IV administration. Most importantly, with these data, the project team could assess the likelihood of this compound achieving therapeutic brain levels in a clinical setting meeting the objectives of determining the drug concentration at the target site. The project team has the option to consider other potential candidates with combined greater permeability and less efflux to increase candidate drug concentrations in the brain parenchyma to achieve required therapeutic levels. Furthermore, using the Pgp knockout model, the team was able to gain greater understanding of the interplay between the two opposing molecular properties of permeability and efflux in controlling entry into the brain. These data serve to calibrate the measured in vitro parameters of permeability and efflux values with the in vivo observation of brain penetration. Further assessment of the interplay of these parameters through simulations such as PBPK modeling can also improve selection of alternative drug candidates.

The MALDI IMS data can also be used to assess the variances observed in earlier homogenate brain to plasma concentration ratios and better design and execute future PK/PD studies. As a standard practice, if there is a difference between the tissue concentrations determined by IMS versus what was observed in previous homogenate LC–MS studies, we will validate the IMS quantification by also performing homogenate LC–MS analysis. Although this requires additional time and effort, validation data build confidence in the project team who typically have less experience and confidence in a “new” technology such as IMS than a more established pharma tool such as LC–MS.

The study described typifies IMS investigations in the discovery stage of the pipeline where the primary driver is to address the three pillars of the drug candidate reaching, engaging, and modulating the target. There may be some variance on whether the IMS study will have a PK focus, as the example presented here includes elements of PD verification or a combined PK‐PD study. These factors will generally be prescribed by the project team depending on how early‐on they are in the discovery cycle or what they prioritize as the major hurdles to project progression. The key element is that the quantitative IMS study provides a “ground truth” regarding the three pillars of the drug candidate and the target. These data provide a basis to calibrate and refine in vitro models, preliminary plasma, or homogenate PK data and modeling.

### 3.3 Delivery systems

The preferred drug delivery strategy relies on the systemic circulation to transport the medicine to the target tissue following oral administration. Although this delivery strategy is well established and convenient for patients, there are several disadvantages that can prevent or limit the use of potentially important therapeutic agents. With oral administration, there must be adequate absorption through the small intestine and minimal first‐pass hepatic metabolism before the drug becomes systemically available to target organs. The loss of drug due to limited absorption or first‐pass metabolism can require higher doses to achieve therapeutic levels of drug to the intended target. However, this also results in an increased drug tissue burden in the GI tract or liver and may result in toxicity to those organs due to reduced safety margins, the difference between therapeutic levels and toxicity thresholds. Intravenous (IV) delivery can avoid these limitations but requires medical professionals for administration for every dose, reducing the convenience and raising the costs. Both oral and IV administration result in all tissues being exposed to the active pharmaceutical agent which can result in off‐target pharmacology or trigger toxicity to the exposure levels used to achieve desired pharmacology at the target organ. Although researchers have long recognized the advantages of delivering an active therapeutic agent to the target site at the exclusion of other organs, only recently have the science and technology made it more feasible.

The concept of using a delivery system to minimize systemic exposure, avoid GI absorption, minimize first‐pass metabolism, and engage the target with a therapeutic dose has been exploited using several strategies. These include (i) a long acting parenteral where an intramuscular injection creates a drug depot that reaches a steady state therapeutic exposure for an extended time period following a single injection,^30^ (ii) encapsulation of the pharmacologically active compound so the drug is only released at the target site, for instance using liposomes,^31^ and (iii) small molecule drug conjugates, systems where the active drug is tethered to a large molecule recognized by the target cell and after binding the conjugate is internalized and the active small molecule is released.^32^ Demonstrating the “selectivity” of the biodistribution in an animal model is a critical milestone for these strategies, which fits well with IMS capabilities.

Advancements in nanotechnology have facilitated the development of a diverse class of nanoparticle‐based drug delivery systems including liposomes. Liposomes are lipid vesicles containing a phospholipid bilayer shell encapsulating an aqueous core that contains the drug payload. As a drug delivery vehicle, liposomes have been shown to be non‐toxic and non‐immunogenic with the potential to accumulate the drug in diseased tissues (e.g., tumor) through the enhanced permeability and retention effect, while limiting exposure to healthy tissue.^33^ Despite spectacular growth in the number of publications on targeted drug delivery using liposomes, the number of clinical successes remains quite small.^34^ Improving the likelihood of clinical success for liposomal drug formulations will require better mechanistic understanding of the regional pharmacokinetics including access to the relevant tissues/cells, payload delivery kinetics, and clearance kinetics.

**FIGURE 8:**
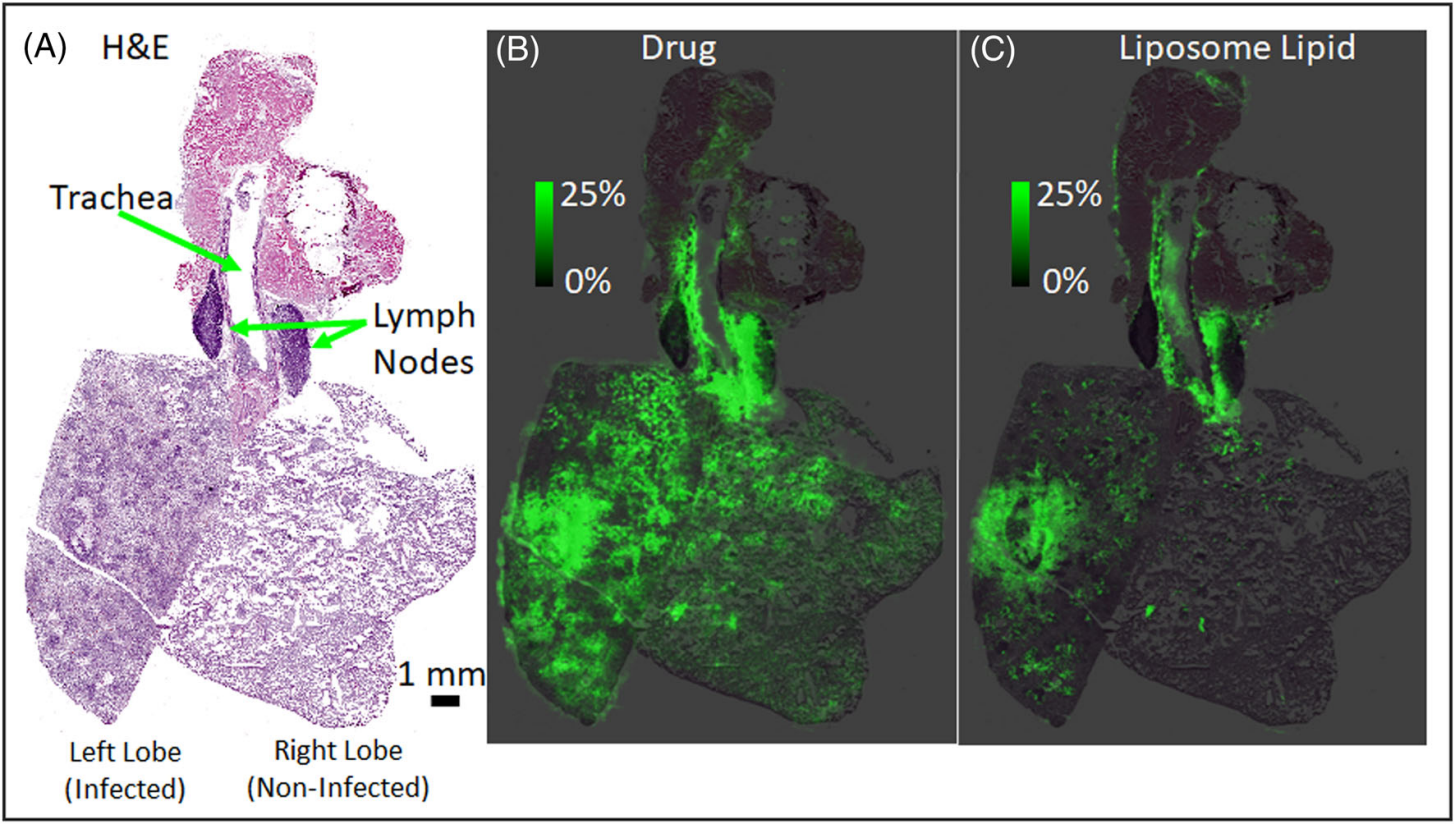
(A) An H&E stained image of the frozen lung where the left lobe has been infected and the right lobe is no infected. Regions of the trachea and lymph nodes are annotated with green arrows. (B) The ion image of the drug scaled from 0% to 25% ion intensity in green overlaid on the H&E image. (C) The ion image of the liposome lipid overlaid on the H&E imaged scaled from 0% to 25% in green

IMS is uniquely suited to deliver valuable insight into the tissue distribution dynamics of these nanocarriers due to its ability to image multiple molecular channels in the same experiment. This capability provides the opportunity to generate molecular ion images of components of the liposomal phospholipid bilayer in addition to the encapsulated drug.^35^ Correlation analysis of the images for the various liposome components can be used to investigate localized regions in a tissue to determine if the liposome remains intact or the drug has been released. For example, Figure 8 displays ion images for a drug (Figure 8b) and liposomal lipid (Figure 8c) in a lung tissue section collected from a mouse given a lung bacterial infection in the left lobe and then administered a single IV injection of an antibacterial drug liposomal formulation. These images, which are from lung tissue collected 24 h post dose, show that the drug localized with highest signal intensity in areas of inflammation and infection surrounding the airways of the infected left lobe as well as within the mediastinal lymph node. The dark purple regions in the infected lobe, seen in the H&E image, are areas of dense inflammatory infiltrate. Lower intensity signals for the drug were observed throughout the non‐infected lobe and trachea tissue. The average intensity of the drug signal was threefold higher in the infected lobe relative to the non‐infected lobe. The distribution of the liposomal lipid in these tissues was similar to the drug, but not identical, with the lipid species appearing to have a more localized distribution than the drug. These observations may indicate that in some areas of the lung tissue, the liposome has been disrupted, releasing the drug to act as a pharmacologically active agent. We have completed several studies over the past few years using MALDI IMS to investigate the tissue dynamics of liposomal delivery, and we see great potential moving forward to help project teams better understand and optimize this delivery platform.

Antibody drug conjugates (ADCs) are a rapidly growing class of oncology immunotherapeutics where a small molecule drug is conjugated to an antibody.^36^ The antibody of an ADC is engineered to specifically target an overexpressed surface receptor on the malignant cells. Upon binding, endocytosis and eventual delivery of the cytotoxic small molecule drug result in cell death. Despite the exciting potential of ADC treatment for cancer patients with over 60 molecules in the clinical development stage, only four ADCs have been approved for use by the FDA and EU.^36^ Imaging technologies can play a critical role in helping researchers better understand how modifications to the components of an ADCs design influence efficacy and safety. For example, Fujiware et al. used MALDI IMS to analyze the intratumor distribution of the anticancer agent monomethyl auristatin E (MMAE) released from an ADC in a xenograft tumor model in mice.^37^ In this work, it was shown that the ion detected was specific to free MMAE without interference from MMAE conjugated to the mAb and therefore could be used to determine the intratumoral release of MMAE from the ADC. This unique ability of MALDI IMS to investigate the distribution of the payload to the desired site of action has the potential to help guide the rational design and optimization of next‐generation ADCs.

The concept of ADCs as delivery vehicles can be extended by replacing the antibody with a “small molecule,” which will bind to the target cell receptor prior to internalization and release of the active compound. This system provides a minimum of three channels to follow the biodistribution and function: (i) the entire conjugate, (ii) the cell binding component, and (iii) the active pharmaceutical agent. Such systems can be explored for proof of concept in both in vitro systems such as 2D and 3D cell‐based systems and in vivo preclinical models.

The efficacy of many pharmacological interventions developed for the treatment of chronic diseases hinges upon maintaining a consistent blood concentration of the drug above a certain threshold for an extended period. For example, modern antiretroviral therapy (ART) for the treatment of HIV is highly dependent upon adherence to a daily combination dosing regimen to maintain systemic exposure of the drugs and achieve viral suppression. The exceptionally high compliance burden this places on patients and the dire consequences of non‐adherence have motivated researchers to explore the use of injectable long‐acting parenteral (LAP; non‐oral route of administration) formulations as a means to provide patients with an option for infrequent dosing regimens (i.e., monthly or quarterly). Injection of a LAP to form a drug depot that can deliver therapeutic drug concentrations over an extended period. Despite the advantages of this approach, the mechanisms driving the dissolution and absorption of LAP drugs are complex, and a better understanding is needed of the impact that the host biological response has on driving drug release kinetics. We recently completed an investigation using a multimodal imaging approach to investigate the temporal evolution of depots of a long‐acting injectable nanosuspension of the viral integrase inhibitor, cabotegravir (CAB). This study included an in vivo imaging portion, where MRI was used to assess the changes in the depot volume over the course of 14 days and an ex vivo portion, where MALDI IMS was used to analyze the localized distribution of CAB within the depot.^38^, ^39^ The MALDI imaging data revealed a high concentration of CAB present in and around the injection site depot at 14 days post‐injection. Additionally, high spatial resolution imaging was used to show CAB localized within multinucleated giant cells and macrophages at the periphery of the depot, suggesting that the infiltration of these species may contribute to the PK observed for CAB long‐acting injectable nanosuspension. This study showed the vast potential of MALDI imaging, as part of a multimodal imaging workflow, to help better understand the highly complex relationship between a drug depot and the surrounding tissue.

Common to all the delivery methods described above is the value of IMS studies to determine the proof of concept and effectiveness of targeted delivery systems for a project team. These data can be an essential part of a large body of data to make go/no‐go decisions with regard to pharmacology, toxicity, and PK. New strategies and the performance of modified engineered materials in vivo can be evaluated against existing systems in a quantitative fashion early in the discovery phase, so only improved methods are further advanced in the pipeline. Some therapeutic targets are amenable to local delivery directly while minimizing concerns of systemic exposure. These include inhalation for drug delivery to the lung, topical formulation applications for dermal targets, and either formulated eye drop solutions or intraocular injections for ophthalmic drug administration. Because of the highly localized drug concentrations achieved by direct delivery to the target tissue, these tissues are also excellent candidates for IMS investigations, including the potential for very high spatial resolution experiments. The further combination of in vivo imaging techniques such as MRI and PET combined with IMS can provide a more holistic view of distribution and performance of selective drug delivery.

### 3.4 Nonclinical safety studies

Safety is a major factor impacting drug attrition throughout the discovery and development pipeline.^40^ Consequently, innovative strategies and technologies to improve the performance of drug candidates through clinical trials continue to be of high importance in the pharmaceutical industry. Similar to the three pillars of PK‐PD and ADME, there has been a focus on building mechanistic understanding and effective decision‐making regarding safety earlier without slowing or limiting the discovery process. Simply collecting more data earlier is not a solution if the data are only used retrospectively after a safety signal is observed later in development. Rather, specifically collecting safety profile data earlier that directs decision making regarding compound selection is the key. Over the years, we have experienced a shift from primarily using MALDI IMS to mechanistically evaluate late‐stage safety issues and risk mitigation to increased investigations of precandidate in vivo safety signals in animal models when the project team feels that a specific drug candidate or target may have development‐limiting toxicity.

Drug‐induced toxicities are generally classified into three types or categories: (i) those directly associated with the target itself (primary pharmacology) where the modulation of the intended target creates the toxicity, (ii) off‐target or secondary pharmacology is the result of an interaction with an unintended target which might be structurally, functionally, or evolutionarily related to the primary therapeutic target, and (iii) those associated with the physicochemical properties of a specific molecule including the formation of reactive metabolites or metabolites, which modulate off‐target sites. The management and mitigation are not prescriptive for each toxicity type, and consideration must be given to the therapeutic area. MALDI IMS studies in the context of safety can help establish a causal mechanistic link between the drug or drug candidate and toxicity, categorize the type of toxicity, provide insight on translation, identify markers, and provide data on risk mitigation. It has been our experience that it is essential to make teams aware of the potential benefits from MALDI IMS studies with respect to safety profiles, so that tissue from planned in vivo nonclinical studies will not be solely formalin fixed or disposed. Because there is a minimum cost associated with collecting some tissue for a MALDI IMS study, we have encouraged project teams who have reason to believe that toxicity might be present in an upcoming in vivo study to flash freeze some tissue for a possible MALDI IMS analysis. If there is no pathology or evidence of toxicity, the samples can be disposed of without conducting the IMS study. On the other hand, if there is toxicity or pathology, the ability to integrate the MALDI IMS tissue analysis with the observed histopathology, clinical chemistry, and clinical observations provide the greatest opportunity to mechanistically assess the toxicity in a timely manner without the burden of repeating the expensive toxicity study.

Early in vivo non‐GLP safety studies in the rodent and non‐rodent models can be used to study dose‐limiting toxicity, including the associated mechanism, identify safety liabilities that could reduce the safety margins, and build a foundation for the drug candidate safety profile. These early studies are conducted at a time where several compounds including different chemical scaffolds may be under consideration. Thus, early safety profiles may be used to direct further chemical synthesis, prioritize drug candidates, or eliminate compounds that have an unacceptable safety profile. For instance, we have used MALDI IMS to profile the safety of compounds that are being “repurposed.” These compounds are often assessed in an early in vivo toxicity study to see if there are any toxicity signals.

**FIGURE 9:**
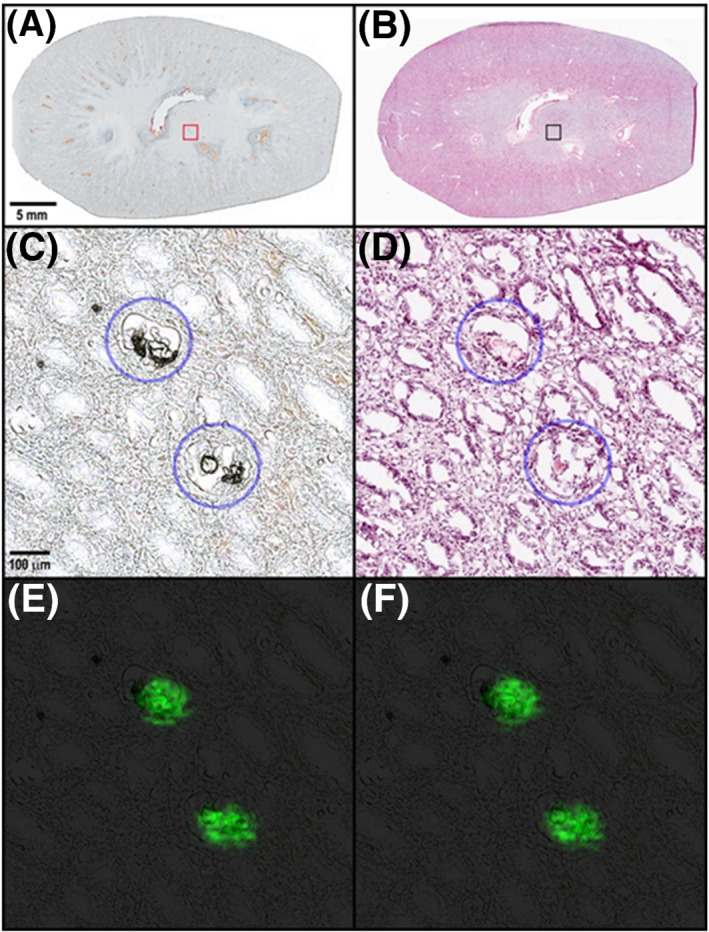
Images of the minipig kidney dosed with a drug candidate as part of a GLP toxicity study. (A) The optical image of the kidney prior to MALDI IMS with a region annotated by the red box for the zoomed in Panel (C). (B) The H&E stained image of the minipig kidney with a region in red indicating the zoomed in figure in Panel (D). (C) The optical image annotates two deposits with blue circles. (D) The corresponding H&E image to Panel (C) where the deposits are no longer visual as a result of the solvents used in the staining process. (E) The ion image of carboxylic acid metabolite. (F) The ion image of the acyl glucuronide M_2_ metabolite

The pivotal GLP toxicity studies establish safety profiles and margins for acute and long‐term exposure of drug candidates based on the therapeutic area. As an example, we investigated kidney tissues from a 3‐month toxicity study in minipigs for an in‐licensed development drug candidate that was targeting stearoyl‐CoA desaturase (SCD) inhibition for the topical treatment of acne. The minipig is often selected as the nonrodent toxicity species in dermal studies, and although the intended administration route for patients was topical, to obtain high chronic plasma exposure levels at multiples higher than in patients, oral dosing of the compound was required for this study. Three dose levels (high, medium, and low) were used in the study so that any toxicity finding may be proportional to the compound exposure. The histopathology assessment of the high‐dose group kidney tissue identified degeneration/regeneration of tubular epithelium and acicular clefts in tubular lumen, suggestive of crystalline deposits of undetermined origin. When sections of the minipig kidney were analyzed microscopically, deposits were not visible in the sections. The kidneys presented small holes throughout, but it was not clear as to the cause of the damage. However, when MALDI IMS was used to analyze the minipig kidney sections, the dosed compound and four metabolites were present in the regions that previously were noted as holes. We concluded that the formalin fixation process dissolved the drug‐related material in the tissue. Deposit compositions in each animal followed a similar trend where a carboxylic acid metabolite (M_1_), formed from oxidation of a benzyl alcohol moiety, was the most abundant ion detected. An acyl glucuronide metabolite (M_2_), the glucuronic acid conjugate of the carboxylic acid metabolite and potential reactive metabolite, was the next most abundant drug‐related species. The deposits also contained lower levels of an ether glucuronide (M_3_; conjugation of the benzyl alcohol), a second carboxylic acid metabolite (M_4_) formed from an amide hydrolysis, and the parent compound. Figure 9 shows the optical image of the kidney (Figure 9A) with a zoomed in region indicated by a red box and the corresponding serial H&E image (Figure 9B) with a black box. The zoomed in regions show the deposits annotated by blue circles in Figure 9C,D. An ion image for M_1_ is shown in Figure 9E and the ion image of the acyl glucuronide M_2_ in Figure 9F. Other metabolites such as M_3_ and M_4_ were detected as well, although with much lower ion intensities. Deposit composition was also evaluated for endogenous species. As no endogenous compounds were detected, it was determined that the deposits were comprised only of drug‐related materials. LC–MS analysis of homogenized tissues detected the same metabolites following the same intensity trends as shown in the MALDI MS data.

The IMS study was a second toxicity study with the goal of better defining the adverse effect dose level and a mechanistic understanding of the adverse drug toxicity in the kidney. The IMS analysis demonstrated that the kidney tubular epithelial disruption observed in the histopathology images was due to drug‐related deposits. It was concluded that the adverse effect was not related to the target or off‐target effects but rather to the solubility (physicochemical properties) of the parent compound and metabolites in the kidney tubules at the adverse effect dose level. If this was the only finding, the safety window or therapeutic window, which describes the difference between the range of therapeutic exposure levels and an adverse effect exposure level, might have been sufficient to safely progress the compound. However, other safety concerns including the lack of a no adverse effect level in the embryofetal development study created a safety risk requiring a much greater safety window than was present. Thus, due to an unacceptable therapeutic window or safety window for the treatment of acne, the project was terminated.

One study highlighting the unique toxicological insight gained by IMS in post‐candidate development is the investigation of dabrafenib in juvenile rats associated with a GLP‐toxicology study.^41^ Dabrafenib is an ATP inhibitor of RAF kinase for the treatment of adult patients with tumors positive for the BRAF V600E mutation. As part of the toxicology assessment for potential use of dabrafenib in pediatric patients, kidney damage was noted in an initial juvenile rat model. That study describes changes such as tubular deposits by microscopy in the juvenile rat models. Because these findings only occurred in the juvenile models and were not present in the adult models or adult patients, it was necessary to understand the distribution of dabrafenib in the juvenile rat kidney and the association with the nephrotoxic response.

**FIGURE 10:**
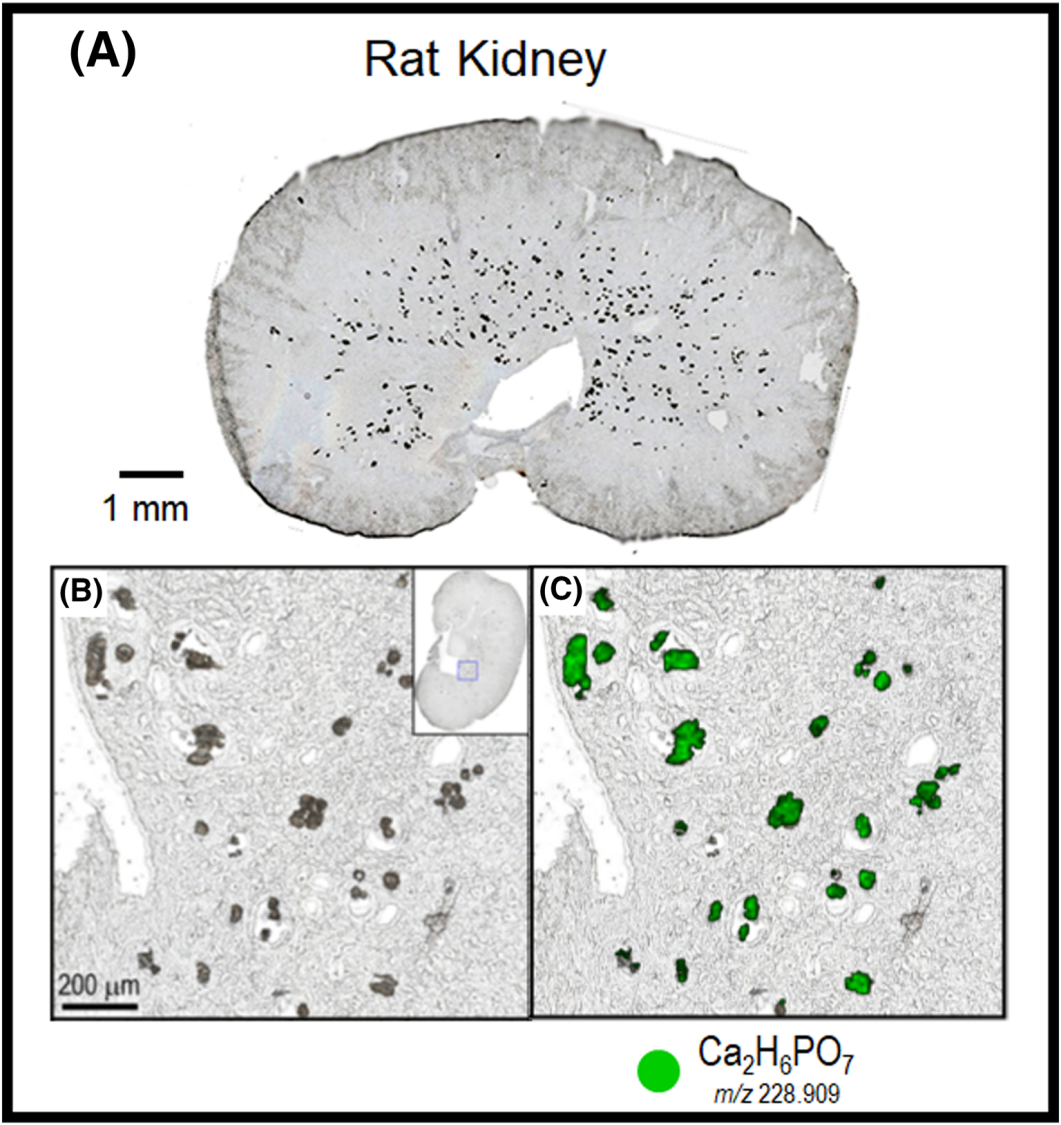
The optical images of a rat kidney dosed with dabrafenib. (A) The optical image with visible deposits in the medulla of the kidney. (B) A zoomed in regions of the deposits visible in the optical image. (C) The same region as in panel B with the ion image of the calcium phosphate ion overlaid in green

Using MALDI IMS, the quantitative distribution of dabrafenib and its metabolites was determined in the kidney sections of various postnatal age groups. Dabrafenib metabolites but not dabrafenib were found to be present in high concentrations in the pelvis and inner medulla regions of the kidney in the youngest age group. However, the distribution of the dabrafenib metabolites did not correlate with the tubular deposits. Furthermore, the concentration of dabrafenib‐related material decreased with the increasing age of the rats, paralleling the kidney nephrotoxicity. Using MALDI IMS and LDI, it was determined that the renal deposits consisted of calcium phosphate clusters. A figure showing the overlay of the calcium phosphate cluster on the optical image is shown in Figure 10A–C. Figure 10A shows the optical image with visible deposits throughout the pelvis and medulla regions. Figure 10B is a zoomed in region of this further showing the deposits. Figure 10C shows the overlay of the ion image with the optical image. It should be noted that the ion image of the calcium phosphate correlates with the deposits shown in Figure 10B.

A concluding hypothesis from this study suggests that immature hepatobiliary elimination in the youngest age group results in high kidney concentrations of dabrafenib metabolites. Damage to the tubular epithelium resulting from the high concentrations of dabrafenib metabolites could trigger localized disruption of the acid–base homeostasis resulting in the formation of calcium phosphate calculi. As the rats mature, the kidney concentration of dabrafenib metabolites and the nephrotoxicity diminish. This study provided unique spatial insights into the molecular mechanism of dabrafenib in the juvenile rat model. The IMS findings in conjunction with the histopathology provided a mechanistic basis for the formation of kidney deposits of preweaning rat pups not observed in adult rats. This information in conjunction with additional preclinical and clinical data allowed the team to make an informed decision about safety risk mitigation in juvenile patients.

Examples of IMS studies providing mechanistic insight into safety issues based on nonclinical IMS studies and powering safety risk mitigation go/no‐go decisions during clinical trials or post‐approval include (i) fosdevirine, an HIV non‐nucleoside reverse transcriptase inhibitor which was linked to seizures in Phase IIb HIV‐1 treatment experience patients due to CNS disposition and metabolism^42^ and (ii) retigabine, an antiepileptic approved drug which was associated with retinal pigmentation changes and discoloration of skin resulting in a blue appearance after long‐term treatment. Retigabine and a metabolite were linked by an IMS study in rats to melanin binding and oxidative dimerization, which was shown to have UV absorbance that gave them a purple appearance.^43^

Through collaborations with pathologists, toxicologists, and project team members, we have established the importance of IMS toxicity studies for understanding safety liabilities, safety margins, the mechanisms associated with a drug‐induced tissue toxicity, development‐limiting toxicity, and toxicity translation. These studies are bespoke, and their success is linked to careful study design, multidiscipline collaboration, as well as the execution of quantitative IMS.

### 3.5 Non‐targeted: An emerging study type

The non‐targeted IMS study designs are the least explored with regard to pharmaceutical discovery and development but have tremendous potential to enhance our understanding of pharmacology, disease pathogenesis mechanisms, and biochemical pathways throughout the pharmaceutical pipeline. The paucity of these studies in drug discovery and development is not tied to the lack of interest or potential but rather the necessity of refined IMS specific statistical tools to analyze the large data sets and identify meaningful spatial changes in tissues. This is an active area of research where analytical strategies and methods are rapidly being developed.^44^, ^45^, ^46^, ^47^, ^48^ Once key molecular ions in images have been identified, the rate‐limiting step is likely to be the time‐consuming process of structure elucidation, which is required in order to derive biochemical meaning and mechanistic interpretation from the observation. Any findings from non‐targeted studies would need to be presented with a high level of confidence if the biological association is to have an impact on the development of a compound. Moving forward the cataloging of molecular ions, their structure, their “normal” tissue distribution, and links to biochemical pathways will become a critical element in advancing this area.^49^, ^50^ The parallel development of spatial transcriptomics and proteomics will enhance specific investigations and promote the development of this area.

In the early stages, we have focused on three elements of non‐targeted study analysis: data reduction methods, spatial segmentation, and a parametric analysis of the segmentation data to determine the spatial specificity of all observed ions to a selected segmentation mask. As statistically based data analysis packages mature, the use of non‐targeted data in pharmaceutical discovery and development will become more routine and transformational.

## 4 PROSPECTIVE … LOOKING AHEAD

In this special feature article, we have highlighted why IMS technology is an important innovation, which can improve and enhance drug discovery and development. It supersedes drug biodistribution approximations or models as the experimental ground truth for quantitative tissue localization. From that vantage point, mechanistic clarity of pharmacology and disease pathogenesis can be gained. Furthermore, translational modeling from preclinical studies can be improved greatly with the input of quantitative IMS data. We have also shared, based on our experience, (i) the types of studies that can be conducted, (ii) strategies and approaches to consider when engaging and collaborating with project teams and other scientists within pharma, and (iii) the importance of driving decision making throughout the pipeline with IMS studies.

We have presented IMS study designs and outcomes that currently make up the largest percentage of effort in pharma (PK‐PD; safety) as well as those we believe will emerge as important study designs in the near future (complex in vitro models; nontargeted pathway analysis). Looking forward, we expect that as the number of completed studies grows so too will our opportunity for data reuse. For example, a drug‐induced liver finding in the rat model will not only use the histopathology images and pathology report to characterize the nature and severity of the findings but will also use the “molecular” signature of the IMS study to compare with previously completed liver studies to provide greater details on the pathways and the stage of pathogenesis. Thus, over time, the IMS data sets we collect will continue to be a valuable resource for understanding all future studies and building knowledge based on a molecular assessment of the tissue. However, our ability to reuse data will require us to establish more rigorous standards for how we acquire our IMS data as well as data curation and experimental protocols.

The continued miniaturization of pharmaceutical science over the past decades, driven by the time and cost associated with repeated synthesis of potential drug candidates for use in assays and preclinical studies, will continue to push analytical detection limits. Furthermore, as drug potency and efficiency increase, we will experience greater demand for improved instrument sensitivity. There also exists greater opportunities to conduct multimodal imaging studies. This is especially powerful when IMS can be coupled with in vivo imaging such as MRI or PET.^38^, ^44^, ^51^ With this combination, biodistribution can be assessed throughout the entire animal model for extended time periods in vivo, with selective terminal time points for more in‐depth biodistribution on the tissue and cellular level by quantitative IMS. The combined data integration then allows scientists to view and understand biodistribution more holistically while minimizing animal use. Similarly, there are greater opportunities to perform multimodal ex vivo imaging studies throughout the discovery and development pipeline.^44^ This includes immunohistochemical (IHC) staining of tissue sections serial to those of IMS in addition to or instead of H&E staining to co‐register with IMS ion images. IHC staining can provide greater tissue differentiation and specificity including cell types or therapeutic target proteins. Furthermore, as more MS‐based cellular imaging develops, these combined MS technologies will be aligned with histological microscopy. The convergence of all these imaging technologies will permit us to examine and explore biodistribution much the same way we use Google Earth© to view a geographical location at multiple levels of magnification. Integrated quantitative imaging platforms will become a framework for all biological and biochemical knowledge in pharmacology and disease pathogenesis.

As highlighted several times in this article, IMS analysis and data mining can benefit from improved analytical tools, databases of molecular tissue distributions, and structural characterization. Furthermore, the current push within pharma for artificial intelligence and machine learning will enhance all aspects of data analysis. However, it is important to remember that all AI/ML‐driven methods, as well as modelling, in drug discovery and development are bound by the quality of the experimental data that they use as input. In this regard, IMS offers an opportunity to establish the ground truth for biodistribution and thus a cornerstone to build on.

Although we have seen the application of IMS within drug discovery and development continue to grow and expand in our organization, we remain focused on delivering tailored studies to answer well‐defined questions, which can drive project decision progression. Integration and collaboration as well as understanding the strategies and requirements for specific therapeutic areas will remain essential for scientists interested in establishing or growing the application of IMS technologies in pharmaceutical or biotechnology environments. Pharma companies will continue to be dynamic work environments that require flexibility on the part of scientists to adapt. The value of any technology and scientists associated with the application of those technologies, including data interpretation, will always be linked to driving successful and timely decision making in the product pipeline.
